# Problems of HIV positive tuberculosis patients' health care in Latvia

**DOI:** 10.1186/1742-4690-9-S1-P69

**Published:** 2012-05-25

**Authors:** Ludmila Viksna, Inga Januskevica, Valentina Sondore, Baiba Rozentale, Ilze Eksteina, Andrejs Ivanovs, Tatjana Kolupajeva, Vija Riekstina

**Affiliations:** 1 Department of Infectology and Dermatology at Riga Stradins University, Riga, Latvia

## Introduction

The number of patients with HIV/TB alliance has increased recently. There were 1055 TB/HIV cases registered in European Union (6% out of 17 650 TB cases tested for HIV) in 2010, among them 71 in Latvia (9.5% out of all TB cases in 2010, in comparison with 0.5% in 2000). The treatment for TB/HIV cases is provided in Latvia according to WHO recommendations, including DOTS strategy. However the TB treatment results for HIV positive cases are concerning due to increasing resistance to HIV/AIDS drugs and to TB drugs.

## Methods

The treatment results for 7761 new smear and/or culture positive pulmonary TB cases were analyzed, 234 among them were HIV positive. In HIV/AIDS naive patients in general the resistance is found in 5.3% of cases. In treated patients the resistance to different groups of drugs is detected in 41% of cases: to all -3%, to NRTI – 14%, to NRTI+NNRTI – 9%, to NRTII+PL – 6%, to NNRTI – 6%, to NNRTI+PI – 1%, to PI – 2%. Multidrug resistance (MDR) was diagnosed in 14% of TB cases. Molecular biological, immunofluorescence, bacteriological and bacterioscopic methods were used for detection of initiating agents. The TB treatment results for HIV positive and HIV negative cases from the period 2000-2007 were compared with the results reported in 2010. Data for 2000-2007 was grouped together due to small number of HIV positive cases within the period.

## Results

The treatment success for HIV positive cases was lower in both periods (60-61%) in comparison with HIV negative cases (78-76%). The level of MDR TB cases among HIV positive patients was higher (15%) than in HIV negative patients (8%) during the years 2000-2007, but similar in both groups (9-10,6%) in 2010. Despite improvements in HIV/AIDS treatment since 2000, the death rate among HIV positive cases in 2010 was higher (18%) than during the years 2000-2007 (11%).

## Conclusions

The resistance to HIV/AIDS drugs is a factor influencing the TB treatment results.

